# Study on the Design and Optimization of a Portable Monitoring and Auxiliary Treatment Device for Upper Extremity Lymphedema–Focus on the Rehabilitation Function of the Device

**DOI:** 10.3389/fbioe.2021.656716

**Published:** 2021-04-01

**Authors:** Xue Yanmin, Zhang Xuyang, Yan Wen, Yu Suihuai, Li Sinan

**Affiliations:** ^1^Department of Industrial Design, Xi’an University of Technology, Xi’an, China; ^2^Industrial Design Institute, Northwestern Polytechnical University, Xi’an, China; ^3^School of Life Sciences and Technology, Xi’an Jiaotong University, Xi’an, China

**Keywords:** upper limb lymphedema recovered, static analysis, wearable design, massage pressure simulation, rehabilitation function of the device

## Abstract

Female patients suffer from the risk of upper limb lymphedema after breast cancer removal surgery. At present, the detection and the adjuvant treatment of this disease are not convenient enough, leading to delay of the disease and increase in the burden of patients. This paper presents a portable monitoring and treatment device for upper extremity lymphedema, enabling patients to monitor the symptoms of upper limb lymphedema and auxiliary rehabilitation. This design utilizes the arm circumference measurement and contrast method to realize symptom monitoring. The device realizes auxiliary rehabilitation using the regional pressure method to imitate traditional manual lymphatic drainage technology. According to the MRI images of volunteers’ upper limbs, the upper arm and forearm’s finite element models are reconstructed in ANSYS. The static simulation experiment is completed. The working mode and parameter design of each rehabilitation module of the device are obtained. The experimental results show that the integrated design principle of monitoring and treatment proposed in this paper has good feasibility, has auxiliary rehabilitation effect, and meets the principle of human comfort. The device can help patients find lymphedema in time and implement auxiliary treatment, which can effectively avoid the further deterioration of lymphedema.

## Introduction

Breast cancer is the leading cancer disease in women. After breast cancer surgery, 15–30% of patients (Chen et al., 2017) in rehabilitation have secondary lymphedema. The cause of the disease was that the lymph nodes draining the patient’s site were also removed at the same time of tumor resection. Therefore, the drainage lymphatic vessel was cut off, which led to the obstruction of lymph reflux in the distal tissue, leading to lymphedema of the upper limbs. Once lymphedema occurs, edema fluid rich in macromolecules remains in the human body’s soft tissue. The human body’s soft tissue will gradually become hard fibrous tissue with fat deposition and proliferation. The patient’s limbs will gradually enlarge and thicken. At the same time, these will cause tissue inflammation. Each infection will aggravate edema, thus forming a vicious cycle. Lymphedema is a chronic disease with slow progress and cannot be completely cured, but if the treatment is timely and there is proper nursing, lymphedema can be obviously relieved.

At present, there is no effective monitoring method for early occult lymphedema. For clinical dominant lymphedema, the following technical methods are generally used for evaluation and measurement: (1) direct measurement of limb circumference or volume, including limb circumference measurement, water substitution method, higher-precision professional equipment Perometer (Shaitelman et al., 2015), etc., (2) bioelectrical impedance analysis equipment is used to indirectly evaluate the degree of swelling by analyzing the body fluid composition of lymphedematous limbs (Kilbreath et al., 2017), and (3) imaging auxiliary examination of lymphedema, including isotope lymphography (Yoon et al., 2020), near-infrared fluorescence imaging (Kraft et al., 2018), and MRI (Lim et al., 2016). At present, the treatment strategy of lymphedema after breast cancer surgery is still in the exploratory stage, and there is no consensus on the treatment of lymphedema. As far as the conservative treatment of upper limb edema is concerned, there are strength training, pressure-assisted therapy, intermittent air wave pressure pump, far-infrared thermotherapy, medication, and so on. These monitoring equipment and auxiliary treatment equipment are set in special nursing institutions or hospitals; patients need to be checked and treated at regular intervals. Patients often miss the best treatment time because they cannot get checked and treated on time for various reasons, resulting in deterioration of the condition. Therefore, it is urgent to develop a wearable monitoring and auxiliary treatment device for lymphedema after breast cancer surgery.

For patients with upper extremity lymphedema, this study can shorten the detection time and increase the convenience of rehabilitation treatment. Compared with the existing clinical diagnosis and rehabilitation treatment technology, this study provides a new and innovative convenient monitoring integrated method and can be used with the existing clinical technology, which will greatly increase the recovery rate of upper limb lymphedema and reduce the delay of rehabilitation treatment due to time. At present, this research belongs to the forefront in China, which will greatly enrich the research field of lymphedema. The device will reduce the pain of patients with lymphedema, achieve the purpose of early detection and treatment, and effectively control the patient’s condition. According to the needs of patients with upper limb lymphedema, this paper designs a portable monitoring and treatment integrated device for upper limb lymphedema and carries out static finite element simulation of human upper limb through ANSYS software, which further optimizes the design of the device, improves the rehabilitation treatment effect of the device, and effectively avoids the further deterioration of patients with lymphedema. Further improvement and application of the device will further improve the living conditions of patients with upper extremity lymphedema and further improve the development of human health.

## Principle of Device Design

In this study, female patients with grade 0 lymphedema after breast cancer surgery (patients with mild or who might have tissue damage) were selected as design users. It can be found that the users lack a device for basic monitoring and auxiliary treatment at home. According to the needs of users, a portable monitoring and auxiliary treatment device for upper limb lymphedema is proposed, which belongs to wearable medical equipment.

### Monitoring and Treatment Principle of Lymphedema of the Upper Limbs

For the portable lymphedema monitoring method, the arm circumference measurement comparison method (Hayes et al., 2008; Czerniec et al., 2010) is more convenient and simple. This method is suitable for household monitoring products because of its low development cost. The arm circumference of the patient was measured regularly every time to detect whether upper limb edema occurred. If upper limb edema is detected, the device will automatically perform the auxiliary treatment function.

**FIGURE 1:**
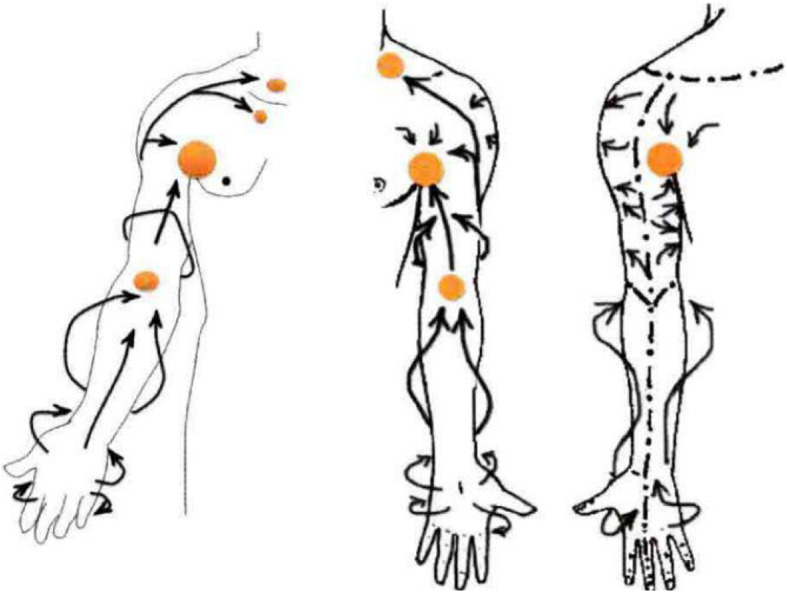
Upper extremity lymph node distribution and manual lymphatic drainage (Ningfei, 2014).

For portable lymphedema rehabilitation methods, the mainstream method is pressure-assisted therapy (Sierla et al., 2018). Because of the gradient pressure difference between the distal end and the proximal end of the limb, pressure will be generated on the lymphatic vessels or blood vessels in the arm. Through uniform pressure bandaging of the whole upper limb, filling, and emptying of lymph and blood can be accelerated, and lymph reflux can be reduced. At present, various elastic bandages are widely used in the market. Another professional mainstream rehabilitation method is manual lymphatic drainage, with the full name of lymphedema manual drainage comprehensive detumescence treatment (Keilani et al., 2020), also known as complete decongestion therapy. At present, this kind of conservative treatment is widely used in the world and has the best rehabilitation effect. Manual lymphatic drainage technology is used to increase or promote the reflux of lymph and interstitial fluid. The implementation method follows the anatomical and physiological pathway of the lymphatic system, which is a professional massage technique. According to Figure 1, in the implementation of the method, the following requirements are essential: massage pressure should be moderate, weak force has no effect, and strong force will lead to lymphatic spasm (Ningfei, 2014; Iannello and Biller, 2020). The speed of force application should be moderate, which is conducive to the smooth change of pressure between tissues in the arm. Each time lasted for 1 to 2 s, and each action was repeated five to seven times. The massage direction should be based on the direction of lymphatic reflux, from the distal end to the proximal end. The massage mode is circular push forward rotary extrusion. The portable monitoring and auxiliary treatment device for upper extremity lymphedema will adopt and combine the above-mentioned detection and rehabilitation methods.

### Principle of Conceptual Design

**FIGURE 2:**
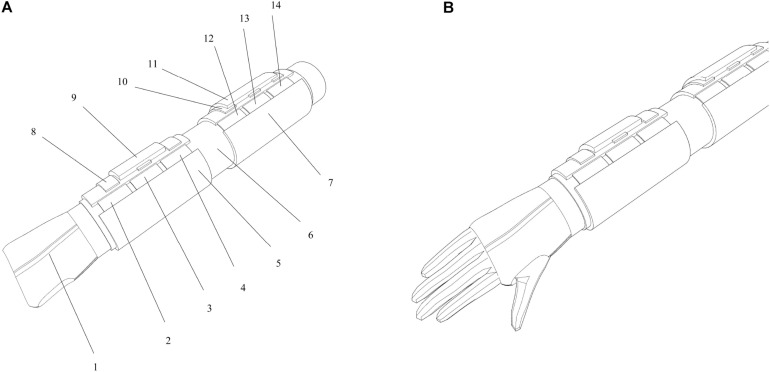
Device diagram: **(A)** schematic diagram of the device structure and **(B)** device wearing diagram.

As shown in Figure 2, the device is based on the use of the pressure-sensing module and the tightening-diastolic module. The device can solve three pain points of users at the same time: how to wear, how to monitor, and how to perform auxiliary treatment. The device is roughly divided into three layers: inner elastic fabric layer (rehabilitation layer: including pressure-assisted therapy), pressure-sensing embedded layer (rehabilitation layer: including manual lymphatic drainage treatment), and external fastening layer (thin rubber, hard cloth, etc.).

The detailed structure of each module is as follows: (1) positioning glove, (2) forearm low-ductility massage belt C, (3) forearm low-ductility massage belt B, (4) forearm low-ductility massage belt A, (5) forearm rehabilitation module, (6) elastic fabric, (7) upper arm rehabilitation module, (8) forearm monitoring module, (9) forearm signal processing module, (10) upper arm monitoring module, (11) upper arm signal processing module, (12) upper arm low-ductility massage belt C, (13) upper arm low-ductility massage belt B, and (14) upper arm low-ductility massage belt A.

The principle of the scheme can solve the problem of inconvenient wearing by one user, monitoring the lymphedema function of the user’s upper limbs, and auxiliary treatment of lymphedema of the upper limbs. Patients can use this device according to their needs after breast cancer surgery.

The principle of the monitoring method is the arm circumference measurement comparison method in portable monitoring and auxiliary treatment device for upper extremity lymphedema. The principle of the device is that the arm circumference measurement module is set in different parts of the upper limb. The user measures the circumference of the same part of his arm every time and compares it with previous data to determine whether the arm circumference changes so as to judge whether the upper limb has lymphedema. Positioning gloves can be used to help in device wear positioning. The principle of rehabilitation is to simulate manual lymphatic drainage treatment and pressure-assisted treatment by mechanical massage. There are two rehabilitation modules in the device that are wrapped in the forearm and the upper arm of the upper limb, which are, respectively, the forearm rehabilitation module and the upper arm rehabilitation module. Each rehabilitation module is provided with three low-ductility massage belts with a width of 50 mm. In the upper arm from top to bottom are the upper arm low-ductility massage belt A, the upper arm low-ductility massage belt B, and the upper arm low-ductility massage belt C. In the forearm from top to bottom are the forearm low-ductility massage belt A, the forearm low-ductility massage belt B, and the forearm low-ductility massage belt C. Each massage belt can apply a fixed output pressure load to each part of upper limb through motor driving or air compression to simulate manual lymphatic drainage.

The subjects of this paper were women aged 20–30 years old with healthy upper limb. The inclusion criteria were breast cancer patients who might have slight upper limb lymphedema after partial mastectomy. The exclusion criteria were other physical defects of the upper limb, such as abnormal growth of lymphatic vessels, partial resection of the upper limb, etc. The middle part of the upper arm and the middle part of the forearm are taken as the rehabilitation reference parts. The simulation of the upper arm low-ductility massage belt B and the forearm low-ductility massage belt B is carried out to improve the parameter design and optimization of the total rehabilitation module of the device. As an excellent finite element analysis software, ANSYS has been more and more used in biological simulation experiments (Nowak et al., 2019; Aubert et al., 2020; Kumar et al., 2020). According to the biological structure of human upper limb, a 3D reconstruction of an upper limb model was carried out, and the simulation results were output by ANSYS software. According to the simulation results, the appropriate force should be applied to the device and how much pressure and energy should be provided by the power module of the device. It provides suggestions for the following detailed design of the device.

## Research on Device Simulation Experiment Based on ANSYS

### Construction of Finite Element Model of Human Upper Limb

**FIGURE 3:**
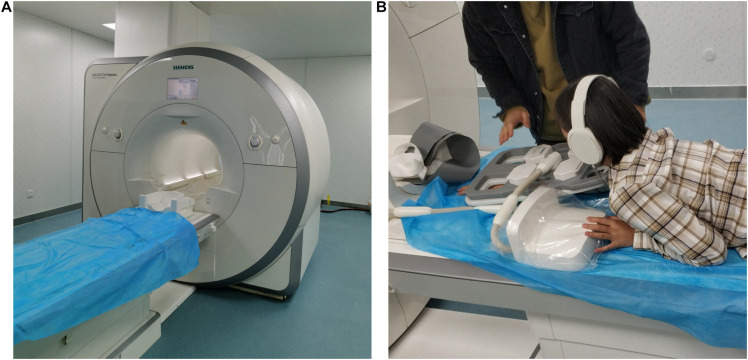
Schematic diagram of human upper limb information collection: **(A)** Siemens MAGNETOM spectra and **(B)** scanned images of a female volunteer.

There are many ways to build the finite element model of biology. Some researchers (Chaojie, 2017) use an MRI scanned image as the original scanning data to simplify and reconstruct the model. In this paper, the scanned image of Siemens MAGNETOM Spectra Magnetic Resonance Imaging System is used as the template to scan the active human upper limb. The middle of the upper arm and the middle of the forearm are used as reference images to simplify and reconstruct the original scanned image. The volunteer for upper limb information collection was a 24 years old urban woman, 165 cm in height and 115 kg in weight. The scanning process is shown in Figure 3.

**FIGURE 4:**
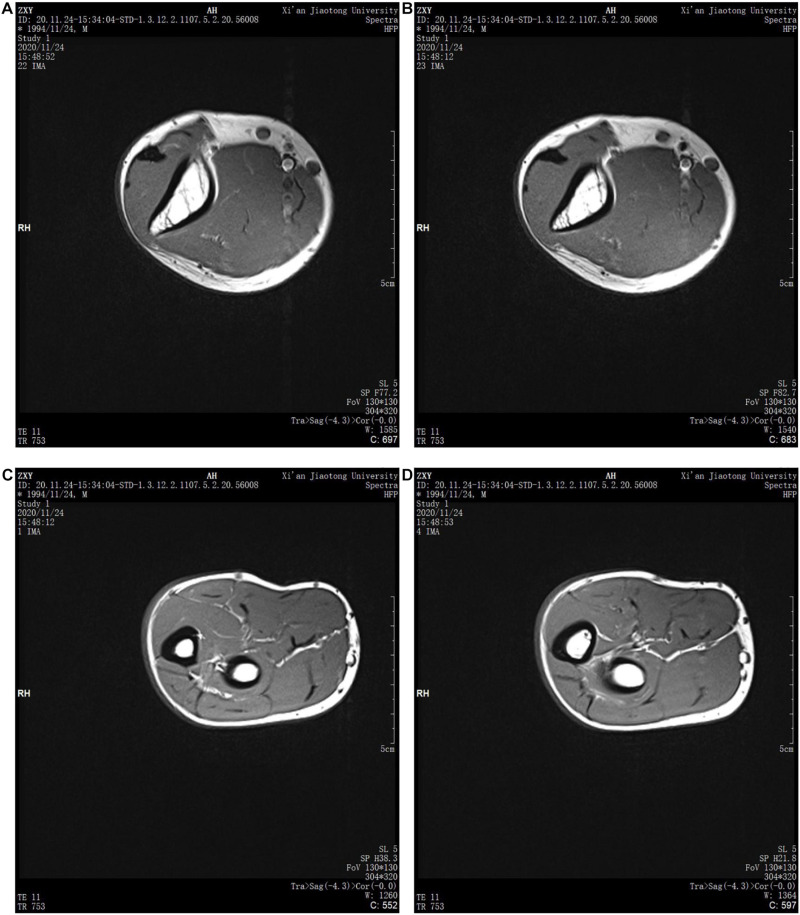
MRI images of human upper limbs: **(A)** upper arm scan Figure 1, **(B)** upper arm scan Figure 2, **(C)** forearm scan Figure 1, and **(D)** forearm scan Figure 2.

Biologically, the cross-sectional structure of the human upper limb is composed of skin, subcutaneous fat, muscle, inner fat, blood, lymph, tissue fluid, and bone. As shown in Figure 4, there are specific gravity differences between the structures of the upper arm and the forearm. In order to obtain accurate experimental data, the upper arm and the forearm should be separated for finite element model reconstruction and simulation experiment.

**FIGURE 5:**
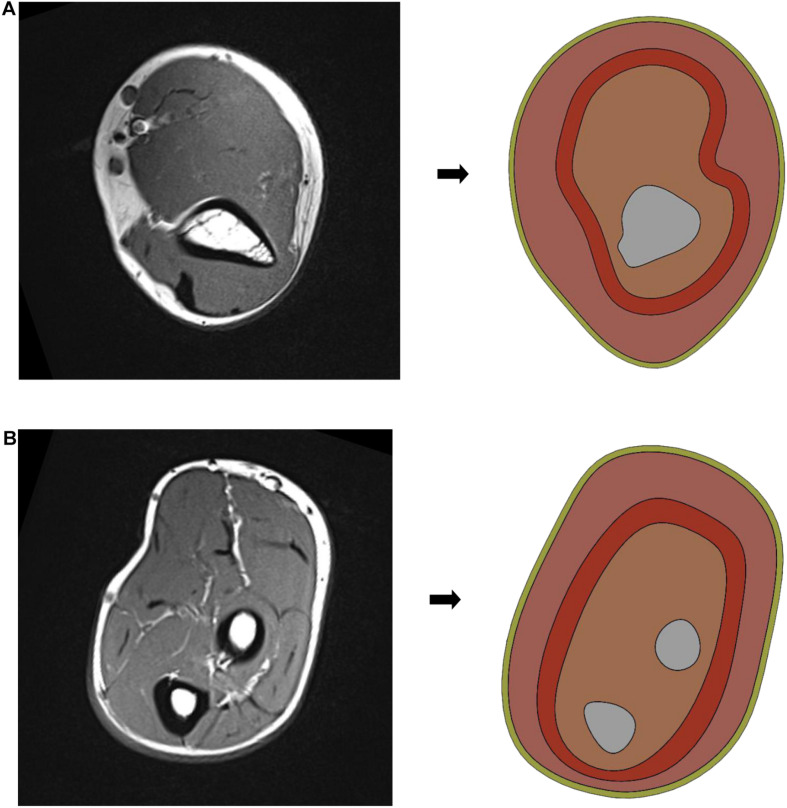
Simplified model of human upper limb: **(A)** simplified schematic of upper arm middle model and **(B)** simplified schematic of forearm middle model.

The human upper arm model was constructed based on the upper limb data of female patients with grade 0 lymphedema (patients with mild or who might have tissue damage). In order to build the finite element model, it is necessary to reasonably stratify the tissues and simplify them according to their similarities and differences, as shown in Figure 5.

**FIGURE 6:**
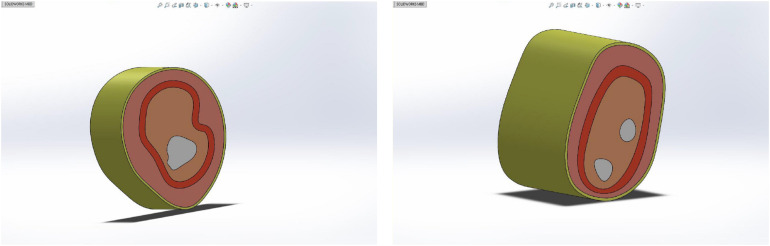
Reconstructed model of upper limb.

In this paper, the structure of the human upper limb is simplified into five parts, which are as follows: bone layer, inner soft tissue layer, lymph and blood layer, outer soft tissue layer, and skin layer. The images of the bone layer were layered obviously and were constructed according to MRI scanned images. Lymph and blood are distributed in the soft tissue layer. Muscle, inner fat, and subcutaneous fat are regarded as soft tissue layer, and they are divided into inner soft tissue layer and outer soft tissue layer by dividing the lymphatic and blood layer. Because the biological structures of lymph and blood are similar due to their physical characteristics, in order to simplify the model, the lymph and blood vessels were divided into a layer with an average width of 4 mm, distributed between the inner and outer soft tissue layers. According to previous research results (Yanning et al., 2008; Jinping and Lianbin, 2020), the width of the skin layer is set at 1.3 mm, and the circumference of the upper arm and forearm is set at 260 and 230 mm, respectively. As shown in Figure 6, in SOLIDWORKS.2016, solid construction of each layer was carried out. The tensile thickness of the middle cross-section of each part of the upper limb model was 50 mm, and it was assembled and saved as X _ T file.

### Mechanical Properties of the Upper Limbs

**FIGURE 7:**
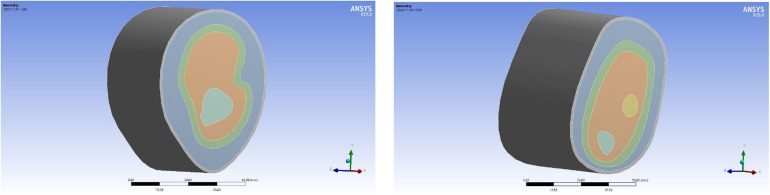
Reconstructed model of upper limb import.

In this paper, static structural module of ANSYS Workbench 19.0 is used for static simulation, and the 3D model built by SOLIDWORKS.2016 in the previous step is imported, as shown in Figure 7. According to the material properties of biological tissues in the relevant literature (Hassan and El-Sheemy, 2004; Lulu et al., 2010; Hong, 2012; Lingpeng et al., 2015; Vanaclocha et al., 2019), the material of each layer of the 3D reconstruction model was reset, as shown in Table 1.

**TABLE 1:** Material properties of upper limb tissue.

Structure	Material type	Density (kg/m^3^)	Elastic modulus (MPa)	Poisson’s ratio
Skin (Hong, 2012)	Elastic	1,000	5.45	0.42
Inner and outer soft tissue (Lulu et al., 2010)	Elastic	1,060	1.5	0.35
Lymph and blood (Lingpeng et al., 2015)	Elastic	1,025	50	0.45
Bone (Hassan and El-Sheemy, 2004; Vanaclocha et al., 2019)	Elastic	1,800	2,084	0.3

### Mesh Generation of the Reconstruction Model

**FIGURE 8:**
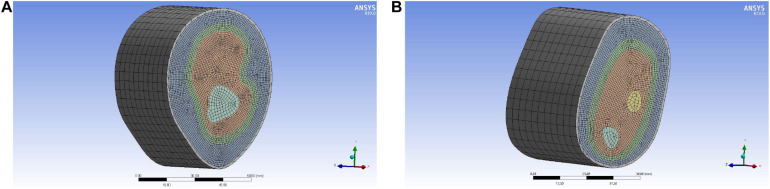
Mesh generation of the reconstruction model of upper limb: **(A)** upper arm model and **(B)** forearm model.

**FIGURE 9:**
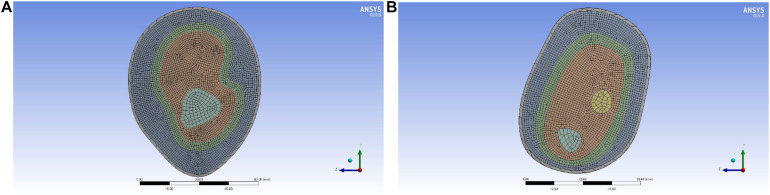
Cross-section of the reconstruction model of upper limb meshing: **(A)** upper arm model and **(B)** forearm model.

In order to get an accurate simulation effect, it is necessary to mesh each layer structure and refine the important parts. The skin layer, as the unimportant part, is set as 5 mm, the outer soft tissue layer, lymph and blood layer, and inner soft tissue layer as important parts had a node size of 1 mm, and the bone layer is 2 mm. The mesh generation of the 3D reconstruction model is shown in Figure 8, and the cross-section of the 3D reconstruction model is shown in Figure 9.

According to the node size divided, the number of elements and nodes of different structural layers in the upper arm model are obtained, as shown in Table 2. According to the node size divided, the number of elements and nodes of different structural layers in the forearm model are obtained, as shown in Table 3.

**TABLE 2:** Unit number and node number in each layer of the upper arm model.

Structure	Node size (mm)	Number of nodes	Unit number
Skin layer	5	1,368	2,990
Outer soft tissue layer	1	343,387	80,631
Lymph and blood layer	1	3,960	550
Inner soft tissue layer	1	169,906	35,598
Bone layer	2	464,199	106,692

**TABLE 3:** Unit number and node number in each layer of the forearm model.

Structure	Node size (mm)	Number of nodes	Unit number
Skin layer	5	3,410	495
Outer soft tissue layer	1	282,046	62,883
Lymph and blood layer	1	156,301	33,048
Inner soft tissue layer	1	272,544	63,546
Bone layer	2	7,647	1,536

### Boundary Condition Setting

The simulation experiment should be similar to the actual massage effect of the device. The boundary conditions are defined as follows: The internal load pressure is applied to the outer surface of the skin layer, and the bone layer is set as fixed. Different pressure was applied to the surface of the skin layer, and the pressure direction was around the skin layer and pointed to the center.

### Solution and Analysis of the Simulation Experiment

In order to promote the reflux of intralymphatic fluid, the pressure in lymphatic vessels should be increased appropriately. The lymphatic research of Olscewski’s team (Olsewski and Engeset, 1980) showed that the pressure of the lymphatic terminal in supine position was 37 mmHg, while standing it was 44 mmHg, and the pressure in the lymphatic vessels was 13.5 mmHg in resting state. In obstructive lymphedema, the pressure in the dilated lymphatics was 0 or slightly higher in supine position, 50–60 mmHg in standing position, and 200–230 mmHg, or even higher, in muscle contraction. According to literature (Avraham et al., 2010), for female patients with grade 0 lymphedema (patients with mild or who might have tissue damage), in order to achieve the rehabilitation effect, the maximum load of the lymphatic tissue layer should be about 0.03 MPa (1 mmHg = 133 Pa), and the overflowing lymph will be squeezed back into the lymphatic vessels.

**FIGURE 10:**
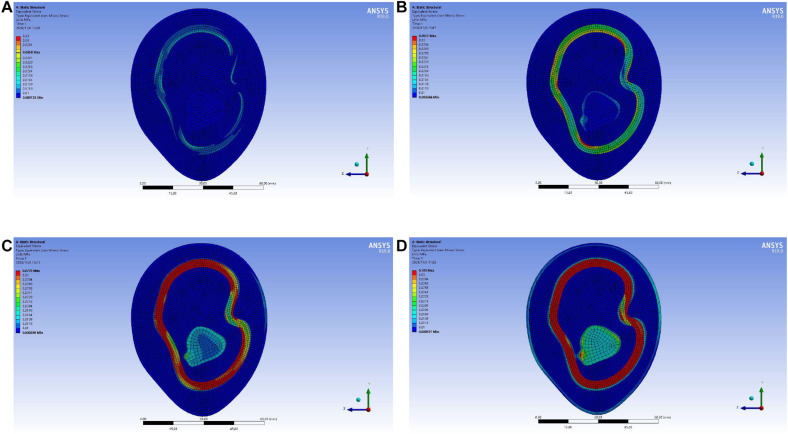
Rainbow diagram of pressure distribution in the lymph and blood layers of the upper arm model under different external pressure loads: **(A)** 002 MPa external pressure, **(B)** 0.004 MPa external pressure, **(C)** 0.006 MPa external pressure, and **(D)** 0.008 MPa external pressure.

**FIGURE 11:**
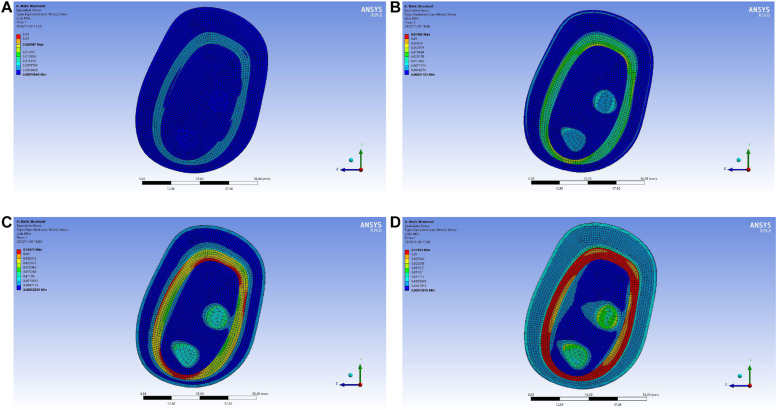
Rainbow diagram of pressure distribution in the lymph and blood layers of the forearm model under different external pressure loads: **(A)** 001 MPa external pressure, **(B)** 0.003 MPa external pressure, **(C)** 0.005 MPa external pressure, and **(D)** 0.007 MPa external pressure.

In the upper arm model experiment, after the continuous test of ANSYS simulation experiment, as shown in Figure 10, the skin layer is continuously applied with a progressive external load. When the loading pressure is 0.002 to 0.008 MPa, the pressure load of the lymph and the blood layer is basically stable within 0.03 MPa. In the pressure rainbow diagram, the red part indicates that the pressure in this area exceeds 0.03 MPa. In the forearm model experiment, after the continuous test of ANSYS simulation experiment, as shown in Figure 11, the skin layer is continuously applied with a progressive external load. When the loading pressure is 0.001 to 0.007 MPa, the pressure load of the lymph and the blood layer is basically stable within 0.03 MPa. In the pressure rainbow diagram, the red part indicates that the pressure in this area exceeds 0.03 MPa.

**FIGURE 12:**
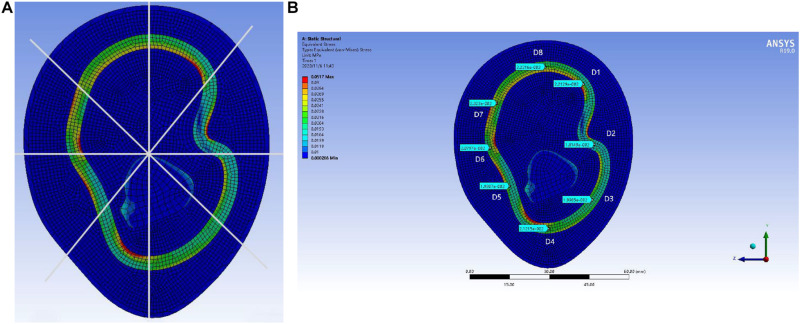
Display of point selection and positioning method of “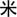-shaped” line mark: **(A)** “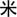-shaped” line mark and **(B)** 0.004 MPa external pressure value of “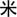-shaped” line mark pressure.

In order to locate the accurate range of external load pressure, the “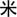-shaped” line mark is used for point selection and positioning, as shown in Figure 12. The center point of the reconstructed model is taken as the origin of the “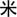-shaped” line. Eight rays from the origin intersect with the midline of the lymph and the blood layers, respectively, and eight intersections are obtained. The eight points were marked clockwise as points D1, D2, D3, D4, D5, D6, D7, and D8. Each point can be selected to obtain the pressure load at this point. In the pressure load diagram of each fixed point, when the values of four random points among the eight points are greater than 0.03 MPa, these indicate that the load of the lymph and the blood layer is too large, indicating that the external load force exerted by the skin layer is too large.

**FIGURE 13:**
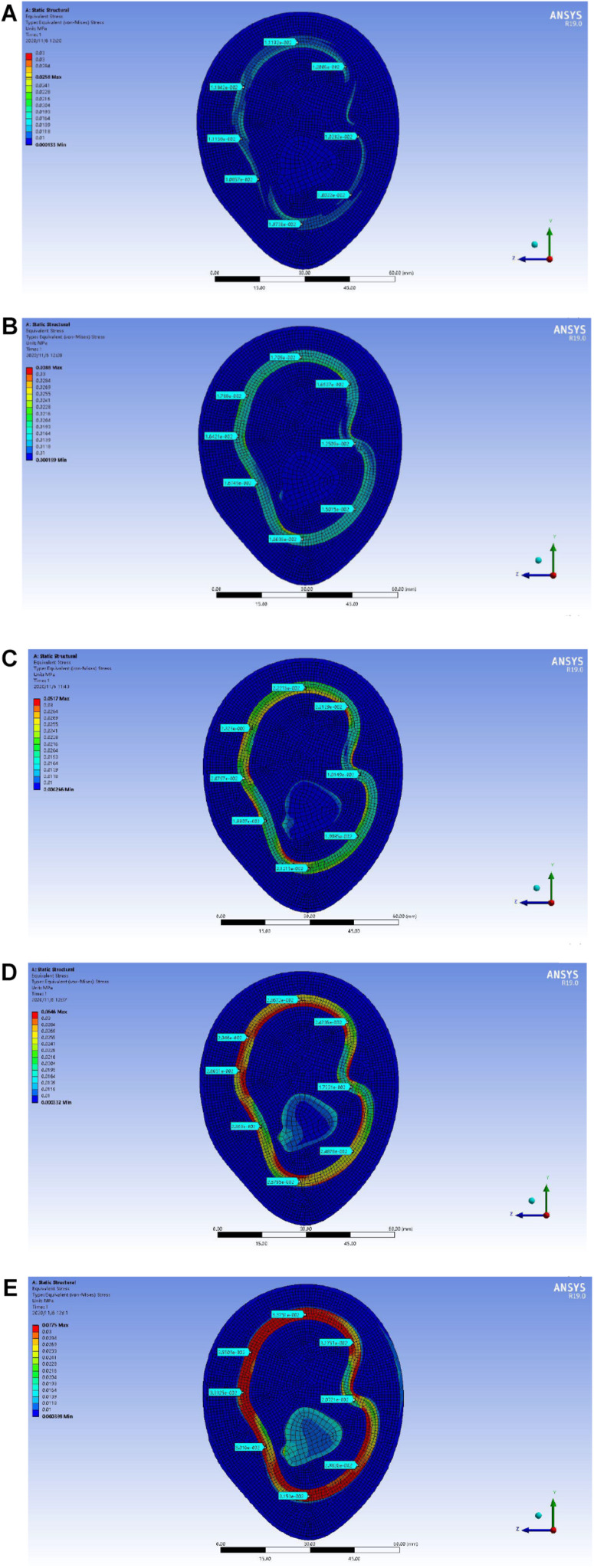
Point selection picture of the lymph and blood layers of the upper arm model with different external pressure loads: **(A)** 002 MPa external pressure, **(B)** 0.003 MPa external pressure, **(C)** 0.004 MPa external pressure, **(D)** 0.005 MPa external pressure, and **(E)** 0.006 MPa external pressure.

**FIGURE 14:**
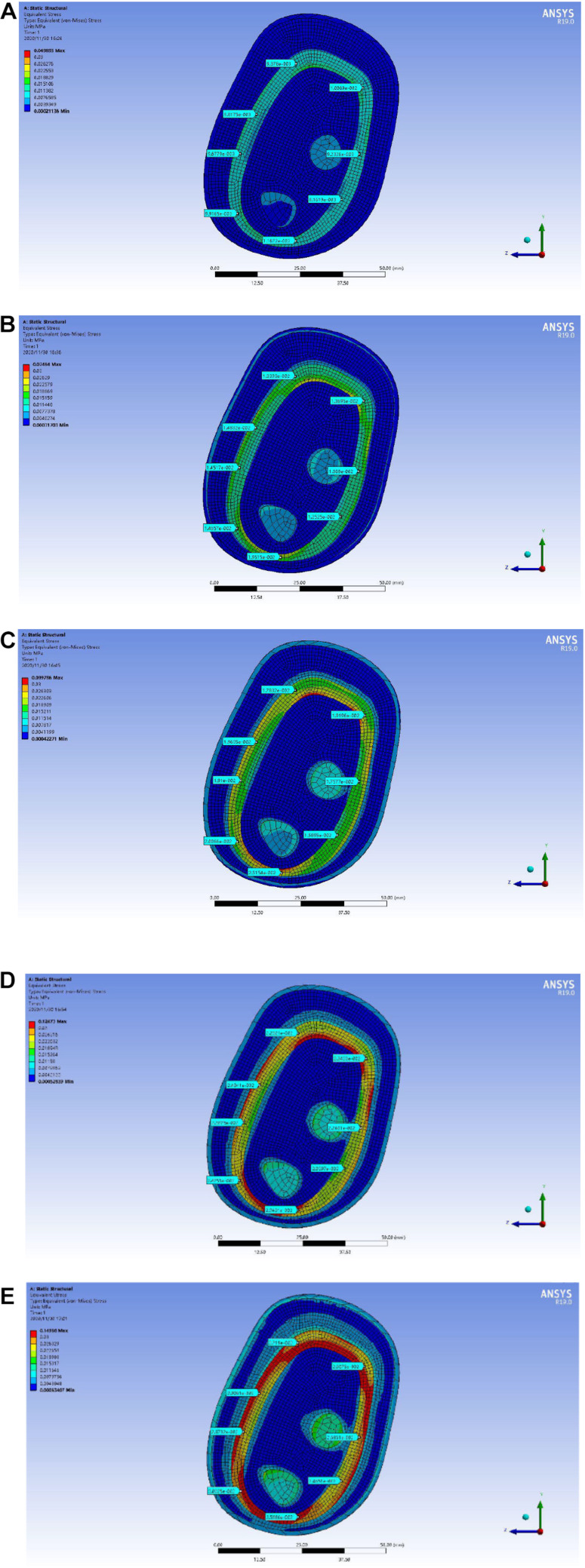
Point selection picture of the lymph and blood layers of the forearm model with different external pressure loads: **(A)** 002 MPa external pressure, **(B)** 0.003 MPa external pressure, **(C)** 0.004 MPa external pressure, **(D)** 0.005 MPa external pressure, and **(E)** 0.006 MPa external pressure.

As shown in Figure 13, the distribution range of the external force and the pressure of the upper arm model is further refined. When the upper arm model is applied with 0.002 MPa external force pressure, 0.003 MPa external force pressure, 0.004 MPa external force pressure, 0.005 MPa external force pressure, and 0.006 MPa external force pressure, the selected point pressure diagram of eight punctuation points is displayed. As shown in Figure 14, the distribution range of the external force and the pressure of the forearm model are further refined. When the forearm model is applied with 0.002 MPa external force pressure, 0.003 MPa external force pressure, 0.004 MPa external force pressure, 0.005 MPa external force pressure, and 0.006 MPa external force pressure, the selected point pressure diagram of eight punctuation points is displayed. The eight point pressure tables of the lymph and the blood layers of the upper arm model with different external force pressures are obtained, as shown in Table 4. The eight point pressure tables of the lymph and the blood layers of the forearm model with different external force pressures are obtained, as shown in Table 5.

**TABLE 4:** Eight-point-specific pressure table of lymph and blood layers of the upper arm model with different external force pressure values.

Applied external force load pressure	Pressure of eight punctuation points in lymph and blood layers (MPa)
0.002 MPa external pressure	D1: 0.012006, D2: 0.010282, D3: 0.010022, D4: 0.010736, D5: 0.010857, D6:0.011158, D7: 0.011842, D8: 0.011192
0.003 MPa external pressure	D1: 0.016937, D2: 0.012508, D3: 0.015015, D4: 0.016636, D5: 0.016749, D6: 0.016421, D7: 0.017680, D8: 0.017090
0.004 MPa external pressure	D1: 0.022129, D2: 0.018149, D3: 0.019985, D4: 0.021215, D5: 0.019907, D6: 0.020797, D7: 0.023230, D8: 0.022216
0.005 MPa external pressure	D1: 0.024236, D2: 0.017331, D3: 0.024878, D4: 0.025795, D5: 0.023630, D6: 0.028651, D7: 0.029460, D8: 0.026672
0.006 MPa external pressure	D1: 0.031751, D2: 0.020721, D3: 0.029826, D4: 0.031530, D5: 0.030160, D6: 0.033325, D7: 0.035509, D8: 0.033751

**TABLE 5:** Eight-point-specific pressure table of lymph and blood layers of the forearm model with different external force pressure values.

Applied external force load pressure	Pressure of eight punctuation points in lymph and blood layers (MPa)
0.002 MPa external pressure	D1: 0.010069, D2: 0.009233, D3: 0.008162, D4: 0.011672, D5: 0.009917, D6: 0.009678, D7: 0.009818, D8: 0.009378
0.003 MPa external pressure	D1: 0.013695, D2: 0.013680, D3: 0.012525, D4: 0.019515, D5: 0.014657, D6: 0.014517, D7: 0.014832, D8: 0.013393
0.004 MPa external pressure	D1: 0.019196, D2: 0.017577, D3: 0.015895, D4: 0.025154 D5: 0.020066, D6: 0.019100, D7: 0.019635, D8: 0.017832
0.005 MPa external pressure	D1: 0.023423, D2: 0.022601, D3: 0.020397, D4: 0.029481 D5: 0.024755, D6: 0.023875, D7: 0.024041, D8: 0.022321
0.006 MPa external pressure	D1: 0.028075, D2: 0.026861, D3: 0.024651, D4: 0.035986 D5: 0.030325, D6: 0.028782, D7: 0.029061, D8: 0.027190

The data obtained by ANSYS software are sorted out and analyzed. In the upper arm model and the forearm model, when the external load pressure of the skin layer is 0.002–0.006 MPa, the pressure range of the internal lymphatic and blood layer is within 0.03 MPa. The experimental results are refined again, and the parameters are optimized according to the design principle of the device.

## Simulation Parameter Analysis and Design Optimization

### Analysis of the Simulation Parameters

In order to verify that the eight punctuation points measured in each picture have a reference value, the 10 groups of data obtained are sorted and analyzed. Shown in formula (1) is the variance formula that was used:

(1) $$S^{2}=\frac{\sum_{i=1}^{n}{\left(x_{i}-x\right)}^{2}}{n}$$

Shown in formula (2) is the standard deviation calculation formula:

(2) $$S=\sqrt{\frac{\sum_{i=1}^{n}{\left(x_{i}-\bar{x}\right)}^{2}}{n-1}}$$

The average pressure load and error range of each group of data were analyzed.

As shown in Table 6, it can be found that, under the same external pressure of the upper limb, the average pressure of the lymph and the blood layer of the upper arm model is slightly higher than that of the forearm. This is because, in human upper limbs, the arm circumference of the upper arm is generally larger than that of the forearm, and the pressure area of the upper arm is larger. According to the formula *F* = *P* ⋅*S*, there is greater force on the upper arm. According to the variance and standard deviation data in Table 6, we can see that there are still some errors in this experiment, but in 10 groups of data, the errors of nine groups of data are within reasonable range, so the experiment has a certain reference value.

**TABLE 6:** Eight-point-specific pressure table of lymph and blood layers of the upper limb model with different external force pressure values.

Applied external force load pressure	Mean pressure (MPa)	Variance (*S*^2^: × 10^–6^)	Standard deviation (*S*)
0.002 MPa external pressure of upper arm	0.0110	0.4225	0.00065
0.002 MPa external pressure of forearm	0.0097	84.456	0.00919
0.003 MPa external pressure of upper arm	0.0161	2.3716	0.00154
0.003 MPa external pressure of forearm	0.0146	3.9601	0.00199
0.004 MPa external pressure of upper arm	0.0210	2.2801	0.00151
0.004 MPa external pressure of forearm	0.0193	6.4516	0.00254
0.005 MPa external pressure of upper arm	0.0251	12.2500	0.00350
0.005 MPa external pressure of forearm	0.0239	6.0516	0.00246
0.006 MPa external pressure of upper arm	0.0308	17.7241	0.00421
0.006 MPa external pressure of forearm	0.0289	9.7344	0.00312

### Optimization Design of the Device Parameters

Through the ANSYS simulation experiment, in the rehabilitation module of the portable upper limb lymphedema monitoring and auxiliary treatment integrated device, aiming at female patients with grade 0 lymphedema (patients with mild or who might have tissue damage), the optimization design scheme of the device rehabilitation module parameters is obtained.

The working mode of the upper arm low-ductility massage belt B in the upper arm rehabilitation module will be set to three pressure ranges: weak grade, 0.003 MPa; medium grade, 0.004 MPa; and strong grade, 0.005 MPa. In order to form a pressure gradient, the pressure at the distal end of the upper limb should be greater than that at the proximal end. The working mode of the forearm low-ductility massage belt B in the forearm rehabilitation module will also be set to the pressure range of the three gears: weak grade, 0.0035 MPa; medium grade, 0.0045 MPa; and strong grade, 0.0055 MPa. The application range of the low-ductility massage belt of the rehabilitation module is 260 mm in length and 50 mm in width. The range of applied force can be obtained.

According to these data, the device parameters in each rehabilitation module are set in detail. In order to achieve the recovery effect of pressure reflux of lymph, it is necessary to adjust the pressure intensity to form a pressure gradient. The working mode values of each low-ductility massage belt of the upper arm rehabilitation module and the forearm rehabilitation module in Table 7 are obtained. The pressure time is set to automatically adjust the frequency, and the pressure time is gradually increased to 1, 2, and 3 s. The starting time of the different grade pressures of each rehabilitation module was not carried out at the same time, and there was a small sequence time. This starts with a low-stretch massage band at the far end of the upper extremity. This will form a better rehabilitation effect; the device will achieve better auxiliary treatment effect.

**TABLE 7:** Set value of working mode of the massage belt in each rehabilitation module.

Rehabilitation module	Massage belt	Weak (MPa)	Medium (MPa)	Strong (MPa)
Upper arm rehabilitation module	Upper arm low stretch massage belt A	0.0028	0.0038	0.0048
	Upper arm low stretch massage belt B	0.0030	0.0040	0.0050
	Upper arm low stretch massage belt C	0.0032	0.0042	0.0052
Forearm rehabilitation module	Forearm low stretch massage belt A	0.0033	0.0043	0.0053
	Forearm low stretch massage belt B	0.0035	0.0045	0.0055
	Forearm low stretch massage belt C	0.0037	0.0047	0.0057

Compared with this device, the traditional detection methods, such as measuring with a tape measure, need the help of others every time and cannot guarantee an accurate measurement of the same marked point of the upper limb; measuring with a measuring cylinder with water displacement method, the measurement error of the patient’s upper limb is great, and the front and rear measurement is cumbersome and inaccurate than using professional equipment, such as Perometer, isotope lymphography, near-infrared fluorescence imaging, etc., Every time the cost is more; each test costs 1,000–3,000 yuan, which ordinary patients cannot afford. For the function of adjuvant therapy, many treatment equipment are time-consuming and expensive; non-surgical treatment methods need long-term and timely treatment, such as strength training, skin care, lymphatic massage, etc., which cost 300–1,000 yuan each time; surgical treatment costs 20,000–50,000 yuan each time, which is particularly expensive, and the patients feel uncomfortable. This device uses arm circumference measurement method, the test results are more accurate and cheap, and the cost of the device is controlled within 2,000 yuan. The auxiliary treatment module can also simulate manual lymphatic drainage, which can replace the traditional rehabilitation treatment method and be used directly at home.

There are some limitations in this paper. We only take the middle part of the upper arm and the middle part of the forearm as the research model, and other parts of the experiment are regarded under the same experimental conditions, but there are still some errors. This device is still in the early stage of development, and there is no good rehabilitation verification effect for different disease stages of upper limb lymphedema. In the future, it will further refine the construction of upper limb finite element model and simulation experiment, further improve the accuracy of experimental data, and provide a more significant auxiliary treatment device for patients with upper limb lymphedema. In this paper, only one female volunteer was scanned, and data were collected. In future research, the database of the upper limb will be enriched and data will be collected and analyzed.

## Conclusion

In this paper, the demand status of female patients with lymphedema of the upper limb after breast cancer surgery was analyzed. Combined with wearable design and detection and rehabilitation of lymphedema, a portable monitoring and auxiliary treatment device for upper limb lymphedema was designed. In order to optimize the parameters of the rehabilitation module of the device, ANSYS software was used for static finite element simulation. By simplifying the biological model of the upper arm and the biological model of the forearm, a 3D reconstruction of the model, reasonable layering and simulation experiments are carried out. After a detailed analysis of the simulation results, the specific parameters of the refining device are obtained according to the simulation results. At the same time, setting the pressure range and working time of the device can further improve its rehabilitation effect. The design of this scheme can solve the problem such that patients with upper extremity lymphedema can complete the process of edema monitoring and adjuvant treatment at home, achieving the purpose of early detection and early treatment and effectively controlling the patient’s condition. This paper makes up for the research content of female patients with upper limb lymphedema after breast cancer surgery and provides a design scheme and optimization method for other researchers.

## Data Availability Statement

The original contributions presented in the study are included in the article/supplementary material, further inquiries can be directed to the corresponding author/s.

## Ethics Statement

Ethical review and approval was not required for the study on human participants in accordance with the local legislation and institutional requirements. The patients/participants provided their written informed consent to participate in this study. Written informed consent was obtained from the individual(s) for the publication of any potentially identifiable images or data included in this article.

## Author Contributions

XY contributed to the methodology and project administration of the study. ZX wrote the original draft of the manuscript and contributed to the product conceptualization and formal analysis. YW contributed to the software and review of the study. YS provided supervision and resources of the study. LS provided experimental equipment and organized the data curation. All authors contributed to the article and approved the submitted version.

## Conflict of Interest

The authors declare that the research was conducted in the absence of any commercial or financial relationships that could be construed as a potential conflict of interest.
